# Remifentanil for sedation and analgesia during awake division of tongue flap in children: a report of two cases

**DOI:** 10.1186/s40981-017-0114-5

**Published:** 2017-08-23

**Authors:** Kaori K. Kuroiwa, Masaaki Nishizawa, Nami Kondo, Haruka Nakazawa, Takanobu Hirabayashi

**Affiliations:** Department of Anesthesiology, Nagano Red Cross Hospital, Wakasato, Nagano, Nagano 380-8582 Japan

**Keywords:** Remifentanil, Tongue flap, Cleft palate

## Abstract

**Background:**

The tongue flap is an accepted treatment method for cleft palate repair. Orotracheal or nasotracheal intubation using a fiberoptic scope is preferred for the division of the tongue flap. We report two cases of tongue flap division in which the patients received adequate sedation and analgesia without tracheal intubation.

**Case presentation:**

Twelve- and 13-year-old male patients were treated at our hospital for tongue flap division, performed as part of a cleft palate repair. We planned to divide the tongue flap under sedation with remifentanil (1 μg/kg/min continuous infusion) and local anesthesia, followed by induction of general anesthesia, and orotracheal intubation after the tongue flap was divided. During the procedure, patients were breathing spontaneously and were cooperative. Patients were able to follow the surgeons’ verbal cues to thrust out the tongue during the procedure, so that the surgeons could easily insert the sutures.

**Conclusions:**

During the division of the tongue flap in two children, excellent sedative and analgesic effects were achieved using continuous remifentanil infusion.

## Background

Since the first report by Guerrero-Santos [1], tongue flap surgery has been used for cleft palate repair [2]. The surgery involves two stages: first, the creation of a tongue flap to close the palatal defect, then the division of the flap and freeing the tongue from the palate. However, the flap between the palate and the tongue may complicate airway management during the second operation. Such an iatrogenic difficult airway is a major cause of concern among anesthesiologists during the induction of anesthesia [3]. Securing the airway before the division of the flap precludes the possibility of excessive bleeding from the edges of the divided flap and aspiration into an unsecured airway. By contrast, securing the airway after the division of the flap prevents the possibility of trauma to the flap, and anesthesiologists can proceed with conventional induction of general anesthesia and orotracheal intubation. Herein, we report two cases in which tongue flaps were divided under sedation without tracheal intubation. Adequate sedation with response to verbal commands was achieved with continuous infusion of remifentanil only.

## Case presentation

### Case 1

**Fig. 1:**
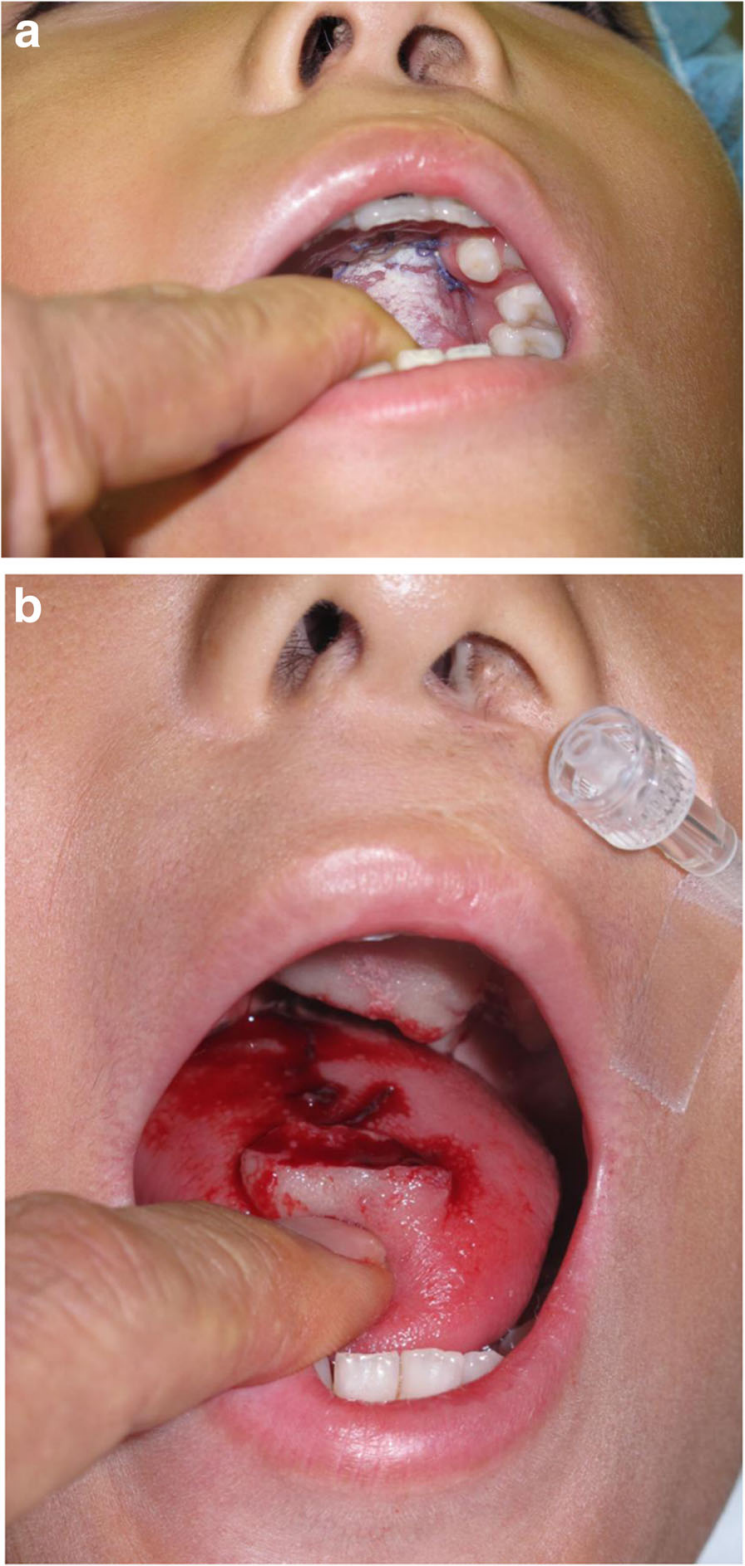
The palate fistula repaired with a tongue flap (**a**) and the flap divided from the tongue (**b**)

A 12-year-old male patient (height, 144 cm; weight, 33 kg) was scheduled for cleft palate repair. His development was normal, and he had no past history, except repeated surgical closure with a mucosal flap for cleft lip and palate. The palate fistula enlarged with his development, and surgical closure with a pedicle tongue flap was scheduled. He received no premedication. General anesthesia was induced with propofol 2 mg/kg, and orotracheal intubation was performed with an I.D. 6.0-mm normal, cuffed tube, using a laryngoscope, following administration of 0.7 mg/kg rocuronium. Anesthesia was maintained with sevoflurane and continuous infusion of remifentanil at 0.2 μg/kg/min. The surgery was completed uneventfully; the palate fistula was repaired with a tongue flap (Fig. 1a). The tracheal tube was removed, in the operating room, after surgery. There were no remarkable changes in respiratory condition postoperatively, although the patient complained of speech disturbances and difficulty eating.

**Fig. 2:**
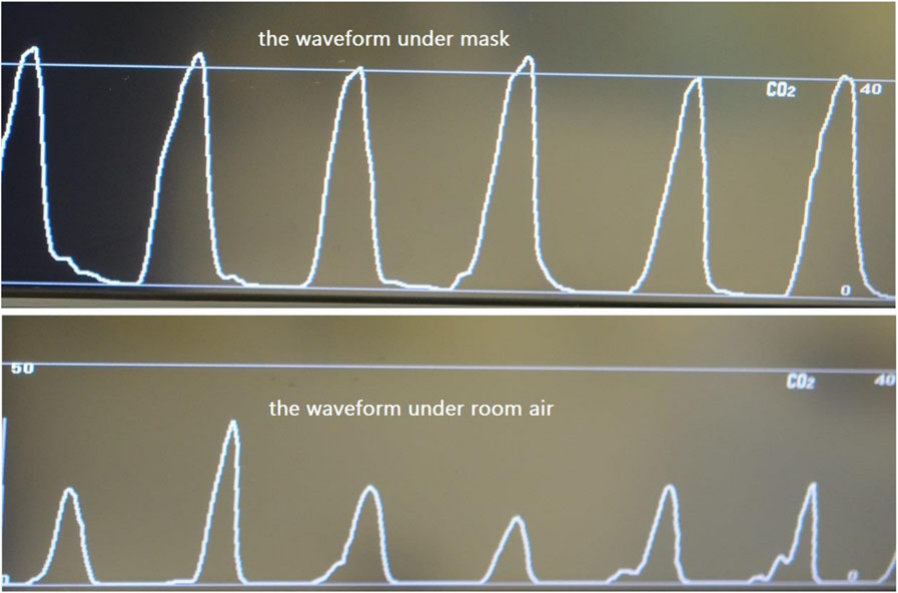
The waveform under the mask and the waveform under room air

One week later, the division of the tongue flap was performed under sedation, exclusively with remifentanil, without tracheal intubation. Under standard monitoring and a capnometer (NIHON KOHDEN, Saitama Japan) with the tip of the sampling tube placed at the side of a nostril to monitor respiratory rate, oxygen was administered at 6 l/min for 3 min. Though he opened his mouth during the procedure, we obtained a respiratory waveform from the capnometer. The waveform under room air was smaller than the waveform under the mask, but we were able to monitor the patient’s respiratory rate (Fig. 2). Then, infusion of remifentanil 1 μg/kg/min was started. He was cooperative and protruded his tongue, in 2 min, in response to the surgeon’s instructions. The patient fell asleep when undisturbed, but responded appropriately to verbal commands, so the sedation level was deemed adequate. The mask was then removed, and local infiltration anesthesia was performed with 0.5% lidocaine and 2 ml of vasoconstrictors at the surgical site in the tongue, followed by a decrease of remifentanil dose to 0.5 μg/kg/min. The flap was divided from the tongue by using a no. 15 scalpel, and the wound was sutured in approximately 5 min (Fig. 1b). Respiratory rate was reduced during remifentanil infusion but remained > 10 breaths/min. Administration of oxygen was resumed, general anesthesia was induced with propofol and rocuronium, and tracheal intubation was performed with a normal cuffed tube using a laryngoscope for surgery to close the palatal fistula. A postoperative interview revealed that he remembered the division with no complaint of pain.

### Case 2

A 13-year-old male patient (height, 142 cm; weight, 31 kg) was scheduled for cleft palate repair. His past and present history were similar to those of case 1. After construction of a tongue flap under general anesthesia with tracheal intubation, the division of the tongue flap was scheduled 1 week later. He was deeply sedated after infusion of remifentanil at 1 μg/kg/min for 2 min, with respiratory rate of 4/min and peripheral capillary oxygen saturation (SpO_2_) of 88%. He responded to verbal commands, and his respiratory rate immediately increased to > 8 breaths/min and SpO_2_ to 100%. The remifentanil infusion rate was reduced to 0.5 μg/kg/min. He was cooperative, and the surgery was performed as described for case 1.

## Discussion

The first problem is how to do of a maintenance of airway. Tongue flap surgery is based on the use of a flap constructed from the dorsum of the tongue to close a defect in the palate [2]. Closure with a tongue flap has the following advantages: formation of a highly vascularized flap, improved flap survival rate even for a large fistula, and extremely low incidence of postoperative disorders of tongue movement, perception, and taste. However, because the tongue flap is a pedicle flap, tongue movement is restricted after tongue flap reconstruction, and restricted movement causes speech disturbance and dysphagia until division. Securing the airway for the division of the tongue flap is concerning for anesthesiologists. Solan [4] suggested to avoid nasal intubation, as this may damage or disrupt the recently constructed flap. But many tongue flap surgery were performed under nasotracheal intubation [5]. Therefore, the ideal approach to secure the airway for the division of the flap would change at the viewpoint of the surgeon and the viewpoint of the anesthesiologist. We believe anesthesiologists should be prepared to safely manage more various approaches. Some reports have described different approaches. Sahoo et al. [6] reported that the division of the tongue flap was performed after direct laryngoscopy and orotracheal intubation under general anesthesia. On the other hand, Peter et al. [7] reported that it was performed under local infiltration anesthesia only. The main difference between the methods is securing the airway either after or before the division of the flap. We completed orotracheal intubation after the division of the flap to avoid damage to the constructed flap.

The second problem is method of sedation and analgesia. Many studies support the usefulness of remifentanil for awake fiberoptic intubation, which requires adequate sedation and maintenance of spontaneous respiration for a short period, as in the cases presented in this report. Several reports describe methods for administering remifentanil to achieve adequate sedation for this procedure, including management with continuous remifentanil infusion from the beginning [8, 9]. We assumed that the initiation of continuous remifentanil infusion at a high dose would prevent adverse events caused by bolus injection and rapidly yield sedative and analgesic effects. Cases in which sedation, adequate for awake fiberoptic intubation, was achieved in adults using continuous remifentanil infusion showed that remifentanil was continuously infused at a maximum dose of 0.5 μg/kg/min [8, 9]. By contrast, Muñoz et al. reported that remifentanil doses required to suppress body movements evoked by stimulation, due to skin incision, were approximately two times higher in children than in adults [10]. Based on these reports, we set the continuous remifentanil infusion dose at 1 μg/kg/min. Although this initial dose was much higher than previously reported doses, we considered it appropriate for rapid achievement of sedative and analgesic effects in children.

The third problem is subsidence by remifentanil. Among the reports on the management of fiberoptic awake intubation using continuous remifentanil infusion in adults, Puchner et al. reported no cases in which SpO_2_ decreased to ≤ 92% [9], whereas Mingo et al. reported that there were one patient with a respiration rate that decreased to 2 breaths/min, and an SpO_2_ that decreased to 78%, and three patients with respiration rates that decreased to ≤ 8 breaths/min, and an SpO_2_ that decreased to 94% [8]; these four patients immediately responded to verbal stimulus, and their SpO_2_ recovered. We also detected similar respiratory depression in the patient described in case 2; the patient immediately responded to verbal stimulus, and respiration rates and SpO_2_ recovered. For safe management during continuous remifentanil infusion, respiratory rates and SpO_2_ should always be monitored while patients are given verbal stimulus. In addition, neither Puchner et al. nor Mingo et al. reported muscle rigidity, respiratory arrest, or hemodynamic fluctuations requiring treatment. Similarly, we did not observe any of these events in our cases. As mentioned in case reports of fiberoptic awake intubation, patients sedated with remifentanil alone are likely to remember the procedure. However, such memories have been reported as rarely unpleasant [8, 9]. In fact, the two patients described in this report, at the postoperative visits, reported that they had no unpleasant memory.

In both of our cases, the possibility of bleeding from the surgical site was extremely low during the division of the flap, because the vascularity of the tongue and flap, as well as the ease of mask ventilation and intratracheal intubation, had been confirmed during tongue flap reconstruction. Moreover, we were ready to perform mask ventilation or intubation immediately if the patients became unresponsive to verbal stimuli and stopped breathing. Furthermore, muscle relaxants were prepared in case of ventilation difficulty due to muscle rigidity.

In our opinion, the method described in the current report allows for sedative and analgesic management without causing detrimental effects, such as respiratory arrest and substantial hemodynamic fluctuations.

The limitation of this method, however, is it was attempted only in cooperative children. When young children do not cooperate during the division of their flaps, anesthesiologists should be prepared for fiberoptic intubation.

## Conclusions

During the division of the tongue flap in two children, excellent sedative and analgesic effects were achieved using continuous remifentanil infusion.

The abstract of this article was presented at the 53rd joint meeting of the Kantokoshinetsu and Tokyo Branches of the Japanese Society of Anesthesiologists (Tokyo).

## Abbreviations

SpO2: Peripheral capillary oxygen saturation
